# Tuberculosis is associated with sputum metabolome variations, irrespective of patient sex or HIV status: an untargeted GCxGC-TOFMS study

**DOI:** 10.1007/s11306-023-02017-7

**Published:** 2023-06-07

**Authors:** Derylize Beukes, Mari van Reenen, Du Toit Loots, Ilse du Preez

**Affiliations:** Human Metabolomics, North-West University, Potchefstroom, South Africa

**Keywords:** Tuberculosis, Sputum, Confounding factors, GCxGC-TOFMS, Metabolomics

## Abstract

**Introduction:**

Various studies have identified TB-induced metabolome variations. However, in most of these studies, a large degree of variation exists between individual patients.

**Objectives:**

To identify differential metabolites for TB, independent of patients’ sex or HIV status.

**Methods:**

Untargeted GCxGC/TOF-MS analyses were applied to the sputum of 31 TB + and 197 TB- individuals. Univariate statistics were used to identify metabolites which are significantly different between TB + and TB- individuals (a) irrespective of HIV status, and (b) with a HIV + status. Comparisons a and b were repeated for (i) all participants, (ii) males only and (iii) females only.

**Results:**

Twenty-one compounds were significantly different between the TB + and TB- individuals within the female subgroup (11% lipids; 10% carbohydrates; 1% amino acids, 5% other and 73% unannotated), and 6 within the male subgroup (20% lipids; 40% carbohydrates; 6% amino acids, 7% other and 27% unannotated). For the HIV + patients (TB + vs. TB-), a total of 125 compounds were significant within the female subgroup (16% lipids; 8% carbohydrates; 12% amino acids, 6% organic acids, 8% other and 50% unannotated), and 44 within the male subgroup (17% lipids; 2% carbohydrates; 14% amino acids related, 8% organic acids, 9% other and 50% unannotated). Only one annotated compound, 1-oleoyl lysophosphaditic acid, was consistently identified as a differential metabolite for TB, irrespective of sex or HIV status. The potential clinical application of this compound should be evaluated further.

**Conclusions:**

Our findings highlight the importance of considering confounders in metabolomics studies in order to identify unambiguous disease biomarkers.

**Supplementary Information:**

The online version contains supplementary material available at 10.1007/s11306-023-02017-7.

## Introduction

Tuberculosis (TB), caused by *Mycobacterium tuberculosis* (*Mtb*), still poses a threat, not only to health, but also to socioeconomics, particularly in low- and middle-income countries. An estimated 10 million new cases of TB, 1.2 million deaths among human immunodeficiency virus (HIV)-negative TB patients, and 251 000 deaths in HIV/TB co-infected individuals were reported in 2018 (World Health Organization 2019). Moreover, TB prevalence differs between males and females, with a male to female ratio of 1.6:1 (World Health Organization 2016).

In the fight against TB, metabolomics has become an increasingly popular research approach. This methodology has been used to investigate various aspects of the diseases, from the basic biology to the identification of disease specific biomarkers and improved treatment regimens (Du Preez et al., 2019). In most of these studies, the cohorts are, however, complex due to inter- and intra-individual heterogeneity (e.g., age, sex, and comorbidities). Many of these internal and external factors can perturb the human metabolome, even in participants considered to be healthy. Darst et al. (2019), for example, indicated that 63,4% (n = 695) of the metabolites identified in healthy human plasma correlated to sex. In an earlier study, a clear variation between the phosphatidylcholine and acylcarnitine metabolite classes were identified in males and females (Li et al., 2018). With regards to HIV, (Cassol et al., 2013), for instance, could differentiate between the untargeted plasma metabolic profiles of HIV patients on antiretroviral therapy and healthy controls, identifying increased bile acids and acylcarnitines and decreased sulfated steroids, polyunsaturated fatty acids, and lysophosphocholine (LPC) in the patient samples, comparatively.

In accordance, the aim of this study was to identify potential variations in the sputum metabolome of TB patients, when including confounding factors such sex and HIV status. The inclusion of these and other covariances into biomarker studies could enable the identification of more sensitive and specific diagnostic and drug targets. Only one annotated compound, 1-oleoyl lysophosphatidic acid, was consistently identified as characteristic for TB, despite the sex and HIV status of patients. According to our knowledge, metabolomics studies done on sputum are limited, and this is the first attempt to investigate these confounding factors in this matrix. Outcomes therefore also suggest improvements to future metabolomics study designs, not only for TB, but for diseases in general.

## Materials and methods

### Sample collection

Sputum samples from patients suspected of having TB, based on a medical assessment of the symptoms associated with the disease were sent to a centralized national laboratory, where standard diagnostic procedures, including both Ziehl-Neelsen staining and bacteriological culture were performed, as per the normal clinical route. After diagnostic testing, the remnant proportion of these samples were frozen (-80 °C) and transported to the North-West University, Centre for Human Metabolomics, for secondary use in research. Samples were received in this manner from March 2009 to May 2010. Anonymity was safeguarded by assigning a unique code to each sample prior to transport. Collected samples were included in the study if they had sufficient volumes for the analyses. Although no additional inclusion or exclusion criteria were considered, some clinical information could be obtained: HIV status (only where the patient requested for an HIV test to be performed), age at time of collection and sex.

### Sample extraction and derivatization

Prior to extraction, 250 µL of each patient sample was homogenized with ethanol (Schoeman et al., 2012) and extracted using a previously described method (Beukes et al., 2019). Samples were randomly analyzed across subgroups, in 19 batches. In short, 50 µL of the internal standard, 3-phenylbutyric acid (0.525 mg mL-1), was added to 250 µL of the homogenised sample. Hereafter, 1 mL of an extraction solvent mixture consisting of chloroform:methanol:water (1:3:1) was added to the tubes (Honeywell International Inc., Muskegon, MI, USA). The extraction was performed using a MM 400 mixer mill (Retsch GmbH & co. KG, Haan, Germany) at a frequency of 30 Hz, for 5 min, after the addition of a 3 mm tungsten carbide bead to each sample tube. Following centrifugation (4 °C at 21 952 x g for 10 min), the supernatant (including the organic and water phases, excluding the pellet) was collected, transferred to a GC-MS sample vial and dried under a light stream of nitrogen. Following this, samples were derivatized using 50 µL of methoxyamine hydrochloride in pyridine (15 mg/mL) (Merck, Darmstadt, Germany) at 50 °C for 90 min, followed by 40 µL of N,O-Bis(trimethylsilyl)trifluoroacetamide (BSTFA) (Sigma-Aldrich, St. Louis, MO, USA) with 1% TMCS at 60 °C for 60 min. The extracts were transferred to a 0.1 mL insert in a sample vial and capped prior to untargeted GCxGC-TOFMS analysis.

### GCxGC-TOFMS analysis

One microliter of each sample extract was injected (1:5 split ratio) onto a Pegasus 4D GCxGC-TOFMS (Leco Corporation, St. Joseph, MI, USA), which comprises an Agilent 7890 A GC (Agilent, Atlanta, GA) coupled to a time of flight mass spectrometer (Leco Corporation, St. Joseph, MI, USA) equipped with a Gerstel Multipurpose Sampler (Gerstel GmbH & co. KG, Eberhard-Gerstel- Platz 1, D-45,473 Mülheim an der Ruhr). First dimensional separation was achieved with a Rxi-5Sil MS primary column (29.245 m, 0.25 mm internal diameter, 0.25 μm film thickness) (Restch GmbH & co. KG, Haan, Germany) and a Rxi-17 capillary column (1.400 m, 0.1 mm internal diameter, 0.1 μm film thickness) was fitted as the secondary column (Restch GmbH & co. KG, Haan, Germany). The front inlet temperature was held at a constant 270 °C for the entire run, ensuring rapid vaporization. For the primary oven, an initial GC oven temperature was set at 70 °C for 2 min followed by an initial increase in oven temperature of 4 °C/min to a final temperature of 300 °C, which was held for 2 min. The secondary column oven temperature was set at 85 °C for 2 min, then increased by 4 °C/min, until a final temperature of 300 °C, at which it was maintained for a further 2 min. The initial temperature of the modulator was 100 °C for 2 min, followed by a 4 °C/min increase to a final temperature of 310 °C held for 9 min. To control the effluent from the primary onto the secondary column, cryomodulation and a hot pulse of nitrogen gas of 0.5 s, every 3 s was used. The acquisition delay for each run was 450 s and the transfer line temperature was held at a constant 270 °C, with the ion source temperature at a constant 200 °C. The detector voltage was adjusted to 1500 V with a filament bias of − 70 eV. Spectra were collected in scan mode from 50 to 800 m/z at an acquisition rate of 200 spectra per second. Note: this was an untargeted approach, with the general aim of separating and detecting as many compounds as possible within a single run (Schoeman et al., 2012, Du Preez & Loots 2013).

Mass spectral deconvolution, peak alignment and peak identification was performed using Leco Corporation’s ChromaTOF software (version 4.51). Mass spectral deconvolution was performed at a signal-to-noise ratio of 100, with a minimum of three apexing peaks. To eliminate the effect of retention time shifts and to create a data matrix containing the relative abundance of all compounds present in all samples, peaks with similar mass spectra and retention times were aligned using Statistical Compare, a package of ChromaTOF. Mass fragmentation patterns and their respective retention times were screened against commercially available National Institute of Standards and Technology (NIST) spectral libraries (mainlib, replib) for peak annotation, with a similarity setting of at least 80%.

### Statistical data analysis

Statistical analyses were performed using MetaboAnalyst (version 5.0) (Pang et al., 2021, R Core Team, 2021). Data were normalized to the sample median, log transformed and auto scaled prior to processing. First, the presence and severity of any batch effect was evaluated, and a summarizing view of the data generated using principal component analyses (PCA). For this purpose, data from all patient samples analysed, grouped according to the batch they were analysed in, were used.

**Fig. 1:**
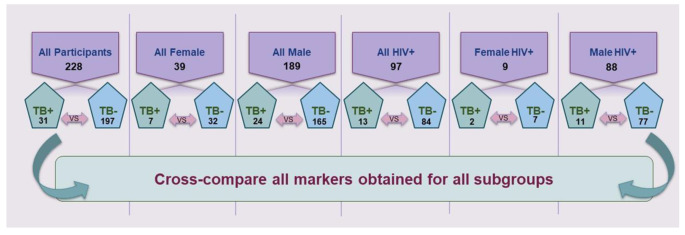
Overview of the composition of the participants in each sample subgroup. Numerical values indicate the total number of participants per subgroup, and double-headed arrows indicate the cohort comparisons used in the statistical analyses

Next, to evaluate the confounding effect of the available clinical information, the processed data were analysed by assigning the participant data to six subgroups, including: all participants (irrespective of HIV status), all female participants, all male participants, all HIV + participants, HIV + female participants, and HIV + male participants (Fig. 1). Based on the classification, individual samples could be added to more than one subgroup for statistical analysis.

Considering the limitation of the study design (i.e. the study was not purposefully designed to assess sex as a main factor), we chose to focus solely on effect sizes as a measure to identify differential TB metabolites. The usual multivariate metabolomics statistical approach is considered inappropriate for small sample sizes. In the univariate setting, the reporting of effect sizes along with p-values from hypothesis testing, has become the new standard. Considering that any findings made here will require further investigation, a nonparametric effect size was used to assess the role of sex in more diagnostic terms. The estimated effect sizes reported here will be invaluable for designing future studies. P-values on the other hand, will be of little value as the current sample size does not allow for the generalization of findings, especially after performing the required adjustments for multiple testing. The probability of superiority (*Aw*) effect size measure was selected due to the qualities it possesses. It is a common language measure which improves the accessibility of the information. That is, the interpretation of the measure is straightforward, it is the probability that a case randomly selected from group 1 will have a higher value than a case randomly selected from group 2. *Aw* makes no assumptions regarding normality or homogeneity of variances and is robust to unequal sample sizes. The R package RProbSup (https://cran.r-project.org/package=RProbSup) was used to compute *Aw* given default settings. Compounds with an *Aw* value exceeding 0.64 between TB + and TB- participants, within each of the subgroups, were shortlisted as informative. An *Aw* value of 0.64 corresponds to a moderate effect. For more information on *Aw*, please refer to Li (2016).

The outputs of the subgroup comparison were then cross-compared to identify those compounds who were identified as differential for TB in all instances. These metabolites were recognized as being characteristic of TB, irrespective of sex or HIV status.

## Results and discussion

### Population

A total of 228 participants, comprising 189 males and 39 females, were included in the study. All participants were adults, aged between 20 and 60 years, with the age range for the male group being 24–60 and 20–58 years for females. The composition of the sample subgroups within the total cohort is stipulated in Fig. 1. Sample sizes (especially the female HIV + group) and the residual spread between TB + and TB- for some of the abovementioned subgroups were very small. As previously stated, the samples were originally collected for diagnostic purposes, and, although this cohort is representative of the typical South African TB patient population, the lack of study design in terms of patient recruitment limits the current investigation. Therefore, it should be noted that this is a preliminary study to understand if sex and HIV-status should be considered as confounding factors in future TB metabolomics investigations. The results are not aimed at identifying set diagnostic biomarkers, as is evident from the selection of only a robust ES to identify differentiating compounds. Expressing metabolic changes in this manner can guide future power calculations to determine appropriate sample sizes, while the identified differentiating metabolites are hypothesis generating, and can be used to guide the choice of future, more targeted analysis. The latter should include MRM-based compound identification and absolute quantification using isotopes and calibrators.

### Overview of the data

In total, 969 features were detected, of which 343 could be annotated by spectral comparison to a commercial library compiled of previously injected standards (with a similarity match of at least 80%). Although the remaining 626 compounds could not be annotated, they were still included in all statistical analyses, and classified as ‘unannotated’. Due to the nature of the classification procedure, some compounds with similar molecular structures and, hence, mass spectra, were given the same annotation (based on the best library hit). Retention times and unique masses were used to determine if these were indeed two different compounds, and if so, the duplicate name was retained with a code, and concentrations analysed as separate compounds. No prominent batch effect or significant gain difference after batch correction was observed (Supplementary info: Figure S1). The dataset was therefore analysed further without applying a batch effect correction procedure.

**Fig. 2:**
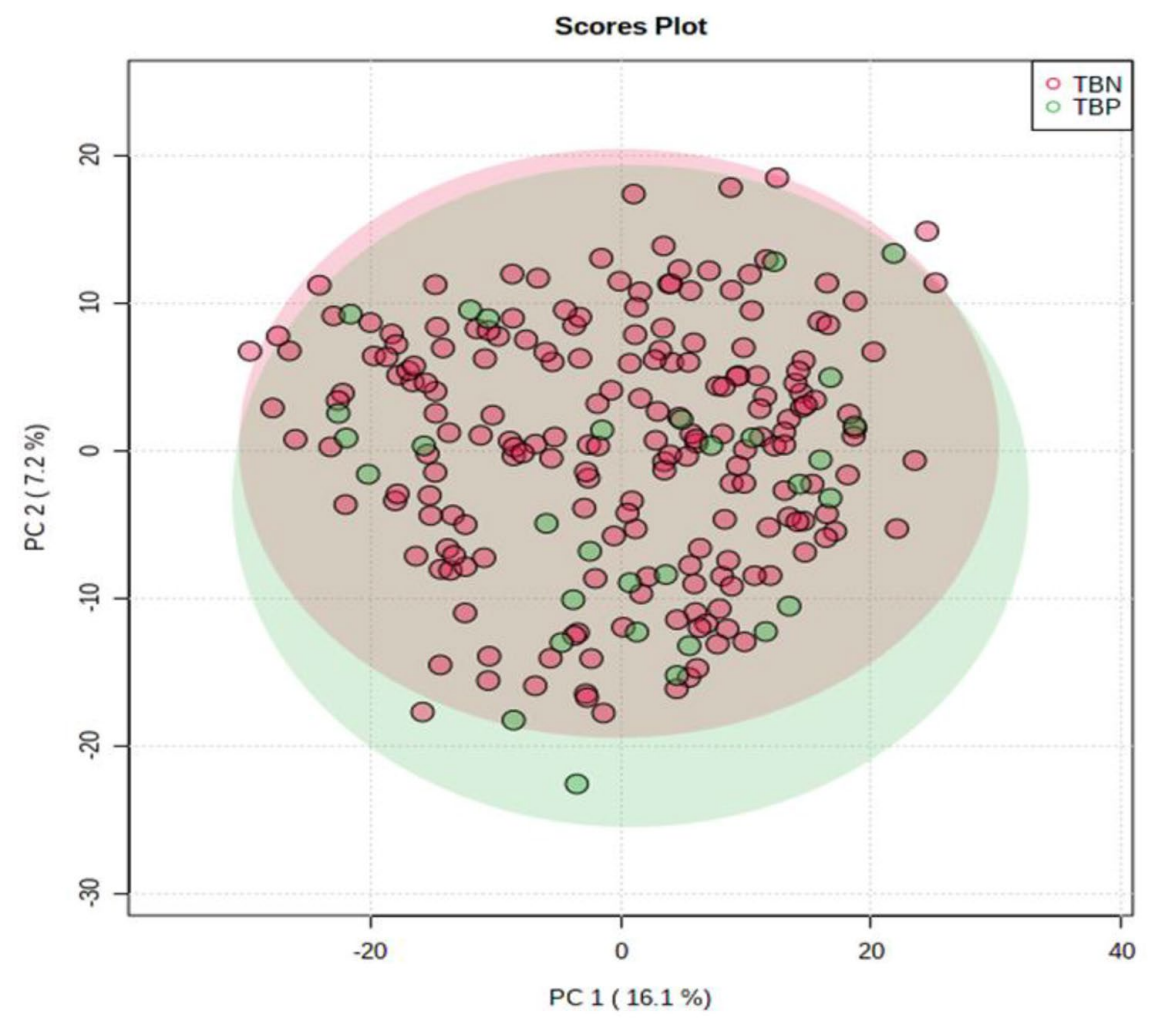
PCA scores plot for complete dataset (all participants), showing no differentiation between the TB+ (TBP) and TB- (TBN) sample groups

Separation between the data obtained from the TB + and TB- individuals, when including all participants, was not evident (Fig. 2). This is consistent with our previous findings (Du Preez & Loots 2013), and can potentially be ascribed to the inter-individual variation in the study population (sex, comorbidities, etc.).

### Subgroup comparisons

**Fig. 3:**
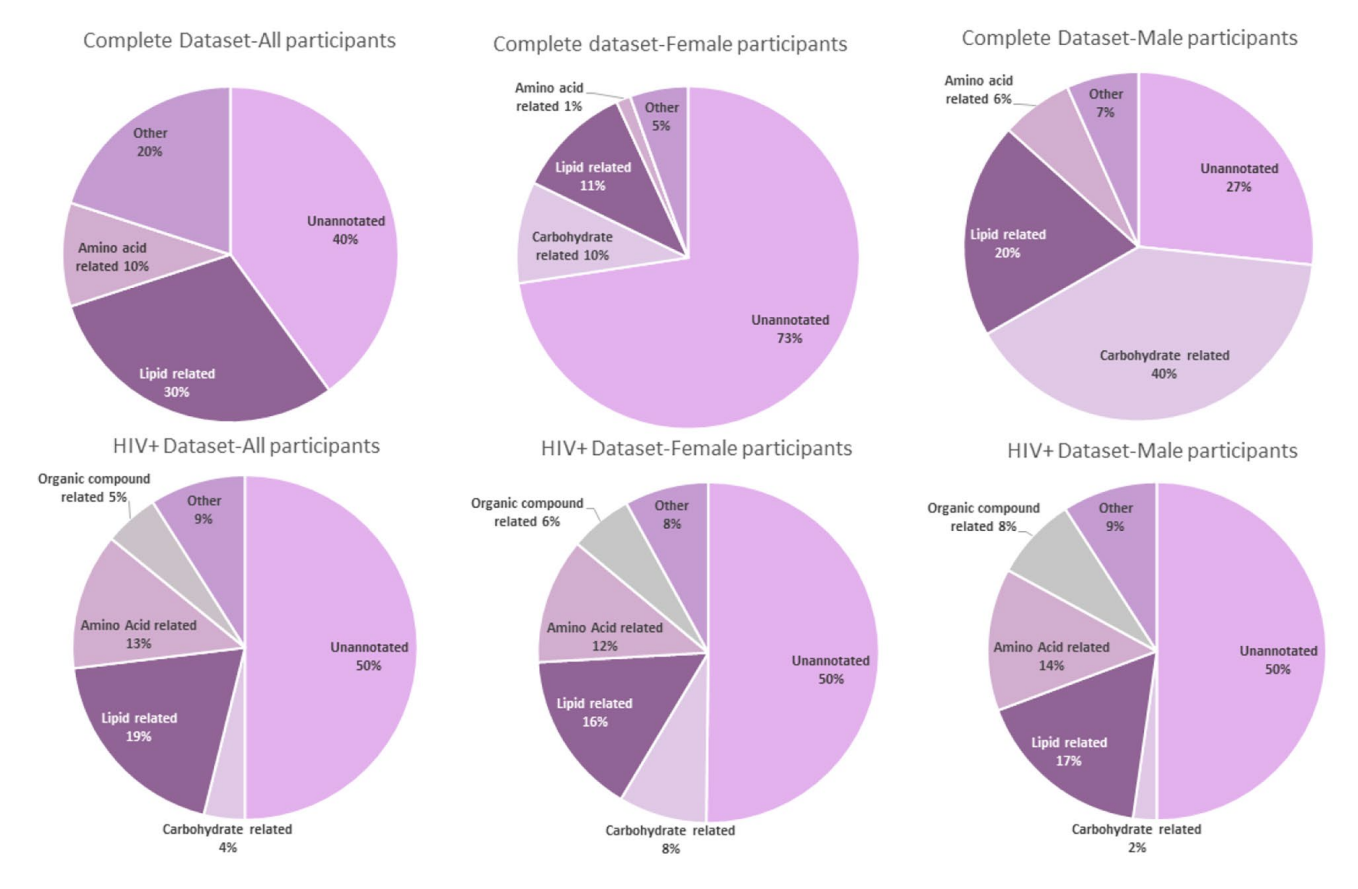
Representation of the number of compounds per class detected with an ES > 0.64 (as a percentage of the total), for each subgroup comparison of TB + vs. TB-.

Details of the statistically significant compounds identified for each subgroup comparison are given in the supplementary info (Table S1 and Table S2). A summary of these metabolic variations, according to the compound classes, is given in Fig. 3. Table 1 stipulates the annotated differential compounds which were either identified in all of the ‘all participant’ subgroups or all of the TB/HIV + subgroups. Most differential compounds in all subgroups were lipids, except in the ‘all male participant’ group, where carbohydrates dominated (Fig. 3). Although the main aim of this study was not to identify gender-specific pathway differences, it is interesting to note that Krumsiek et al. (2015) previously determined that the entire super-pathway of carbohydrates (specifically including glycolysis, gluconeogenesis, pyruvate, fructose, mannose, galactose, starch, and sucrose metabolisms) was significantly higher in the serum of healthy males, compared to females.

#### Differential TB compounds: all male and female participants

When including all participants (Table S1), 10 compounds were identified as significantly different when comparing the TB + and TB- groups, of which 6 could be annotated. In the female subgroup, 74 significant compounds (21 annotated) were identified, including mostly lipids and carbohydrates, of which 8 were also differential when including all participants. In the male group, 10 compounds were significantly different between the TB + and TB- participants, and all 6 of the annotated compounds were also differential in the ‘all participants’ group. Only 4 compounds (3 annotated) were constantly detected as differential when considering all three ‘all participant’ subgroups, including glycine, 2-hexadecanone, and 1-oleoyl lysophosphatidic acid (LPA) (Table 1). Although these compounds has not yet been identified as sputum TB markers, glycine has been detected as a marker in serum (Zhou et al., 2013) and 1-oleoylglycerophosphocholine (lysoPC(p-18:1(9Z)), a direct precursor of 1-oleoyl LPA, was identified as part of a TB serum biosignature (Feng et al., 2015).

#### Differential TB compounds: HIV + male and female participants

A total of 104 (39 annotated) compounds, mostly lipids and amino acid related, were identified as differential when including all HIV + participants (Table S2). Furthermore, 337 (125 annotated) and 163 (44 annotated) compounds were differential in the HIV + female and HIV + male subgroups, respectively. Twenty-five compounds were significant when cross-comparing differential compounds identified for the three HIV + subgroups (Table 1), indicating that TB has a more prominent effect on the metabolism in HIV + individuals, compared to a population where HIV is less frequent (‘all participant’ groups). Interestingly, two metabolites, including heptadecanoic acid and 2,5-dimethoxy-4-(n)-propylphenethylamine, were identified as differential for TB in both the female and male HIV + groups, but with opposite trends. Since the variations between sexes were not the main aim of this study, this was not investigated further.

Although various studies have applied metabolomics as a tool to investigate different aspects of HIV, using diverse sample matrices, metabolomics studies exploring the TB/HIV co-infection are scarce (Liebenberg et al., 2021). In 2019, Silva et al., indicated that arachidonic acid and the glycerophospholipid metabolism were altered in the plasma of HIV patients with paradoxical TB-associated immune reconstitution inflammatory syndrome (IRIS), when compared to non-IRIS TB/HIV patients (Silva et al., 2019). In the current study, we also identified both arachidonic acid and 1-oleoyl LPA, which is an intermediate of the glycerophospholipid metabolism, as differential in all HIV + subgroups (Table 1). Two other studies have identified changes in the tryptophan/kynurenine ratio when comparing HIV + and HIV- TB-patients (Adu-Gyamfi et al., 2017; Collins et al., 2020). Although we detected six amino acid related compounds characterising TB in the HIV + subgroup, neither tryptophan nor kynurenine were included in this list.

#### Cross-comparison of all markers identified for all subgroups

Two compounds, of which one could be annotated as 1-oleoyl LPA, were identified across all subgroup comparisons (Table 1). Although the second compound (hereafter referred to as compound X) could not be annotated based on the set criteria of a similarity match > 80%, its mass spectra could be denoted as oleic acid, with a similarity match of 76,4% (Supplementary material Figure S4). Boxplots of compound X in all subgroups are provided in the supplementary material (Figure S2). This compound showed an overall decrease in the TB + cases compared to TB- individuals when including all participants, but an increase in the HIV + patients. In addition, compound X was higher in the TB patients in both the male subgroups, compared to the TB- cases, but this trend was reversed in both female groups. The standard deviations do, however, indicate large variation between the individuals within the subgroups, and therefore, no valid conclusions can be made from these results without further investigation.

**Fig. 4:**
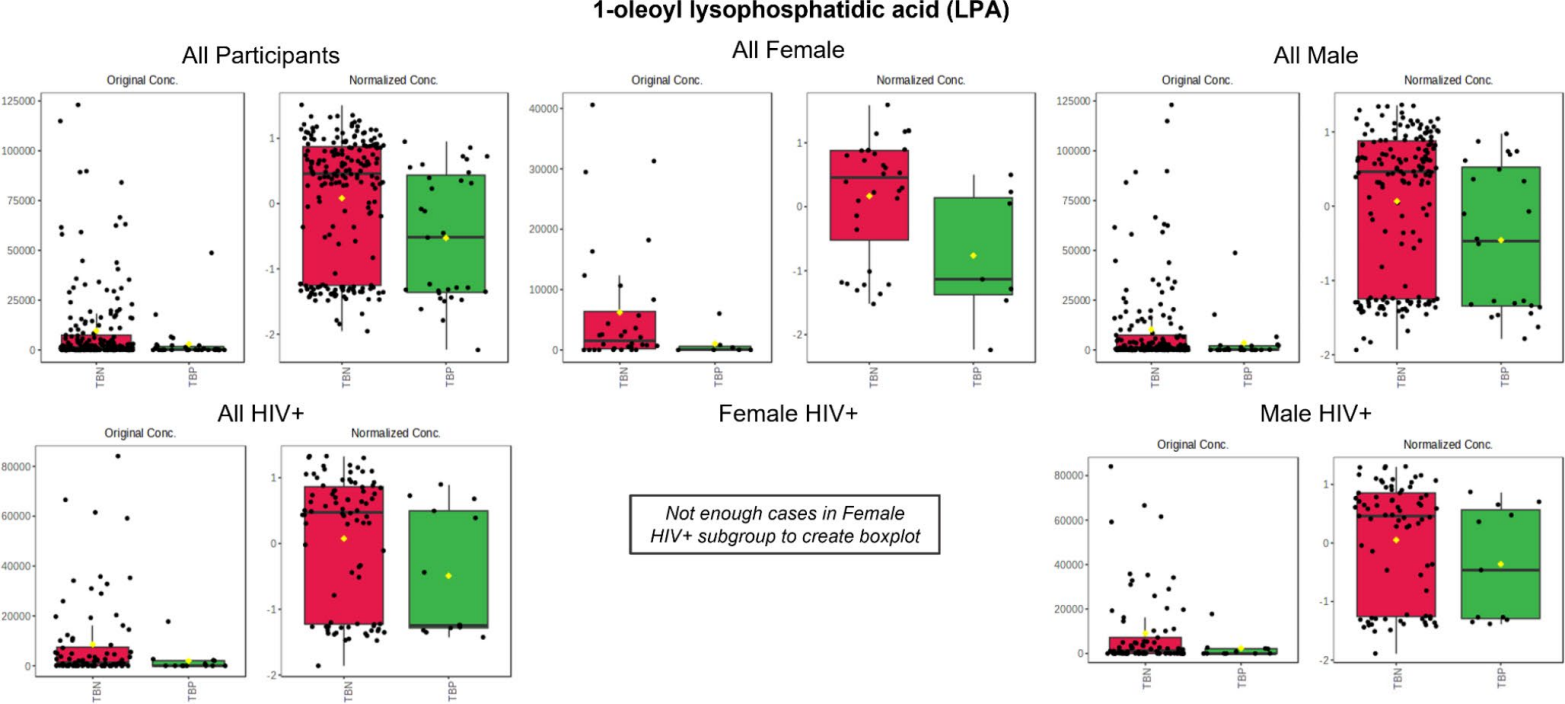
Boxplots of the compound, annotated as 1-oleoyl lysophosphaditic acid (LPA), in all of the subgroup comparisons, compiled using the original concentrations and normalised concentrations. Due to the small sample size of the female HIV + group, boxplots could not be created

Boxplots of 1-oleoyl LPA, in all subgroups, are provided in Fig. 4. These results indicate a significant difference between the TB + and TB- participants in each subgroup. It should be noted that the annotation of this compound was based on a comparison of its mass spectrum to those in a spectral library, which showed a similarity match of 82.4% compared to the spectrum of 1-oleoyl LPA (supplementary material Figure S3). The compound’s mass spectrum was also closely related to that of 1-margaroleoyl LPA acid and 1-palmitoleoyl LPA with similarity matches of 76.5% and 74.0% respectively (supplementary material Figure S4). We will therefore discuss this outcome in terms of LPA in general, irrespective of the fatty acid chain length.

Previous studies have described changes to the lipid metabolome (lipidome) in TB patients (Han et al., 2021; Chen et al., 2021), including our own on sputum (Du Preez & Loots 2013; Schoeman et al., 2012). In mice, *M.tb* uses fatty acids as the main source of carbon (Ghazaei, 2018) and host lipids are the predominant nutrient source for these infective bacteria (Han et al., 2021). Our current study highlights the potential role of LPA in the pathogenesis of TB and TB/HIV co-infection. LPA is a bioactive phospholipid, consisting of a glycerol backbone with a hydroxyl group, a phosphate group, and a fatty acid chain, produced during the synthesis of cell membranes. LPA is described as an important extracellular signalling molecule present in all eukaryotic tissues and blood plasma. These compounds also play an active role in modulating and inducing cell proliferation and migration, not only during cell development but also in pathological conditions. In some cancers, LPA signalling has been linked to the biological events triggering the development of therapy resistance or responses to treatment (Geraldo et al., 2021).

Various studies have specifically linked LPA to lung pathologies. When investigating pulmonary fibrosis in humans, it was shown that LPA-LPA receptor 1 (LPA-LPA1) signalling plays a critical role in the progression of the disease (Tager et al., 2008). Furthermore, during lung injury, increased levels of LPA are produced in bronchoalveolar lavage. The LPA in turn induces the accumulation of fibroblasts and vascular leakage via LPA_1_, advancing the progression of fibrosis (Aikawa et al., 2015). A study monitoring the plasma lipid levels of TB patients from initial diagnosis until cured, indicated that some LPAs show promise as biomarkers for TB. They added that the intervention of lipid metabolism could potentially block energy metabolism and in turn inhibit the cell wall synthesis of *Mtb* (Chen et al., 2021).

A metabolomics study by Weiner et al. (2012) compared TB patients to healthy participants. They found that serum amino acids, medium-chain fatty acids, and the LPA precursor, LPC, were in lower abundance in the patients, comparatively. In addition, the metabolic profile of active TB disease, compared to latent infection, showed decreased phospholipase activity. Phospholipase A_2_ (PLA_2_) hydrolyses the ester bond at the sn2 position of membrane phospholipids, usually resulting in the release of free fatty acids and lysoglycero-phospolipids, the latter of which are precursors of LPA. PLA_2_ is essential for inducing inflammation and plays a key role in the immune response (Wesley Burks et al., 2019). Accordingly, in 2020, Han et al. (2021) applied ultra-high-performance liquid chromatography-tandem mass spectrometry to investigate plasma lipid levels in patients with TB, lung cancer, community-acquired pneumonia, and healthy controls. The study found decreased plasma phospholipid levels (LPA: bioactive phospholipid) and speculated that *Mtb* infection might be responsible for regulating the lipid metabolism of TB patients by promoting host-assisted bacterial degradation of phospholipids (Han et al., 2021). Lower abundances of LPCs/LPAs in TB patients could mechanistically be related to the induction of macrophage apoptosis by *M. tuberculosis* through inhibition of PLA_2_ (Duan et al., 2001).

Coincidently, a significant difference in plasma LPA concentration attributing to sex and age was identified amongst 100 healthy individuals (Michalczyk et al., 2017). However, when investigating sex as a confounding variable in the sputum metabolomes of lung cancer patients, no significant effect was observed, indicating that the lung pathology-induced variation in LPA overshadows the effect of sex (O’Shea et al., 2016).

The findings of this study highlight the need for an in depth targeted lipidomics study to thoroughly investigate and understand how lipids, and LPA, contributes to the pathology of TB. The identification of specific LPAs, such as 1-oleolyl LPA in sputum, additionally presents an opportunity for potential biomarker discovery or profiling for diagnosing TB in males or females with or without HIV co-infection. The exact identity of this compound should, however, be confirmed using compound standards and more sensitive MS methods such as MS/MS and ion mobility. In addition, the absolute quantification using isotopes and calibration curves, in a larger cohort, recruited from different endemic countries, would be essential in the validation process.

**Table 1:** Annotated compounds with ES > 0.64 in either all the ‘all participant’ subgroups, or all the HIV + subgroups

		Total cohort						HIV+					
Class	Compound*	All Participants		Female		Male		All Participants		Female		Male	
		Effect size	Decreased (↓) or increased (↑) in TB + group	Effect size	Decreased (↓) or increased (↑) in TB + group	Effect size	Decreased (↓) or increased (↑) in TB + group	Effect size	Decreased (↓) or increased (↑) in TB + group	Effect size	Decreased (↓) or increased (↑) in TB + group	Effect size	Decreased (↓) or increased (↑) in TB + group
Carbohydrate related	Glucose (UM:204)	-	-	-	-	-	-	0.66	↓	0.68	↓	0.67	↓
Carbohydrate related	Inosine (UM:230)	-	-	-	-	-	-	0.67	↓	0.86	↓	0.65	↓
Lipid related	1-Palmitoyllysophosphatidic acid (UM:299)	-	-	-	-	-	-	0.71	↓	0.86	↓	0.69	↓
**Lipid related**	**1-Oleoyl Lysophosphaditic acid (UM:357)**	**0.66**	**↓**	**0.73**	**↓**	**0.64**	**↓**	**0.67**	**↓**	**0.86**	**↓**	**0.64**	**↓**
Lipid related	Cholesterol (UM:129)	-	-	-	-	-	-	0.75	↓	0.93	↓	0.74	↓
Lipid related	Eicosane (UM:57)	-	-	-	-	-	-	0.71	↓	0.93	↓	0.68	↓
Lipid related	Heptadecanoic acid (UM:117)	-	-	-	-	-	-	0.66	↓	0.64	↑	0.66	↓
Lipid related	à-Linolenic acid (UM:67)	-	-	-	-	-	-	0.67	↓	0.79	↓	0.65	↓
Lipid related	Arachidonic acid (UM:80)	-	-	-	-	-	-	0.69	↓	0.79	↓	0.67	↓
Lipid related	Oleamide (UM:144)	-	-	-	-	-	-	0.66	↓	0.64	↓	0.66	↓
Lipid related	Palmitaldehyde, dibutyl acetal (UM:57)	-	-	-	-	-	-	0.67	↓	0.79	↓	0.64	↓
Amino acid related	Glycine (UM:102)	0.69	↓	0.68	↓	0.70	↓	-	-	-	-	-	-
Amino Acid related	L-Ornithine (UM:142)	-	-	-	-	-	-	0.65	↓	0.64	↓	0.65	↓
Amino Acid related	L-Valine (UM:144)	-	-	-	-	-	-	0.68	↓	0.79	↓	0.67	↓
Amino Acid related	DL-Ornithine (UM:174)	-	-	-	-	-	-	0.69	↓	0.79	↓	0.66	↓
Amino Acid related	4-Coumaric acid (UM:293)	-	-	-	-	-	-	0.68	↓	0.79	↓	0.65	↓
Amino Acid related	2-Aminomalonic acid (UM:218)	-	-	-	-	-	-	0.67	↓	0.64	↓	0.66	↓
Amino Acid related	N-à-Acetyl-L-Lysine (UM:174)	-	-	-	-	-	-	0.69	↓	0.64	↓	0.71	↓
Organic compound related	Ethanolamine (UM:174)	-	-	-	-	-	-	0.65	↓	0.71	↓	0.65	↓
Organic compound related	Parabanic acid (UM:243)	-	-	-	-	-	-	0.68	↓	0.75	↓	0.67	↓
Organic compound related	2,5-Bis((trimethylsilyl)oxy)pyrazine (UM:241)	-	-	-	-	-	-	0.64	↓	0.79	↓	0.64	↓
Other	Decanamide, N-(2-hydroxyethyl)- (UM:85)	-	-	-	-	-	-	0.68	↓	0.79	↓	0.66	↓
Other	Ethylone (UM:144)	-	-	-	-	-	-	0.66	↓	0.68	↓	0.67	↓
Other	Oxazole, 2-(8Z)-8-heptadecen-1-yl-4,5-dihydro- (UM:98)	-	-	-	-	-	-	0.68	↓	0.71	↓	0.66	↓
Other	2,5-Dimethoxy-4-(n)-propylphenethylamine (UM:174)	-	-	-	-	-	-	0.65	↓	0.68	↑	0.65	↓
Other	3-Chloroamphetamine (UM:116)	-	-	-	-	-	-	0.65	↓	0.71	↓	0.66	↓
Other	2-Hexadecanone (UM:58)	0.69	↓	0.73	↓	0.68	↓	-	-	-	-	-	-

*UM* unique mass

## Conclusion

This is the first study to show that confounding factors can result in major differences in the metabolome’s response to *M. tuberculosis* infection. Covariates such as sex and comorbidities should be considered when using metabolomics to explore disease mechanisms. This approach could assist in the development of improved study designs, and therefore, the identification of more specific diagnostic and treatment procedures for TB.

## Electronic supplementary material

Below is the link to the electronic supplementary material.

Supplementary Material 1

Supplementary Material 2

## Data Availability

Raw data were generated at the Centre for Human Metabolomics, North-West University, South Africa. Data supporting the findings of this study are available from the corresponding author on request.
