# Supplementary material for: Tuberculosis is associated with sputum metabolome variations, irrespective of patient sex or HIV status: an untargeted GCxGC-TOFMS study
Source: Metabolomics. 2023 Jun 7;19(6):55. doi: 10.1007/s11306-023-02017-7 (PMC10247825; doi:10.1007/s11306-023-02017-7)
Supplement: Supplementary file 2 — Supplementary Material 2 [file 11306_2023_2017_MOESM2_ESM.docx]

**Supplementary info to:**

**Tuberculosis is associated with sputum metabolome variations, irrespective of patient sex or HIV status: An untargeted GCxGC-TOFMS study**

**Abbreviated title: Differential TB sputum metabolome**

Derylize Beukes, Mari van Reenen, Du Toit Loots, Ilse du Preez*

Centre for Human Metabolomics, North-West University, Potchefstroom, South Africa

***Corresponding author:**

Ilse du Preez

https://orcid.org/0000-0001-5456-3931

Email: ilse.dupreez@nwu.ac.za

Telephone: +27 018 285 2535

Derylize Beukes: [13128531@nwu.ac.za](mailto:13128531@nwu.ac.za) https://orcid.org/0000-0003-1987-4480

Du Toit Loots: [dutoit.loots@nwu.ac.za](mailto:dutoit.loots@nwu.ac.za) https://orcid.org/0000-0002-0339-6237

Mari van Reenen: [12791733@nwu.ac.za](mailto:12791733@nwu.ac.za) https://orcid.org/0000-0002-5856-3258

**Note: Tables S1, S2 and S3 are included as a supplementary Excel workbook.**


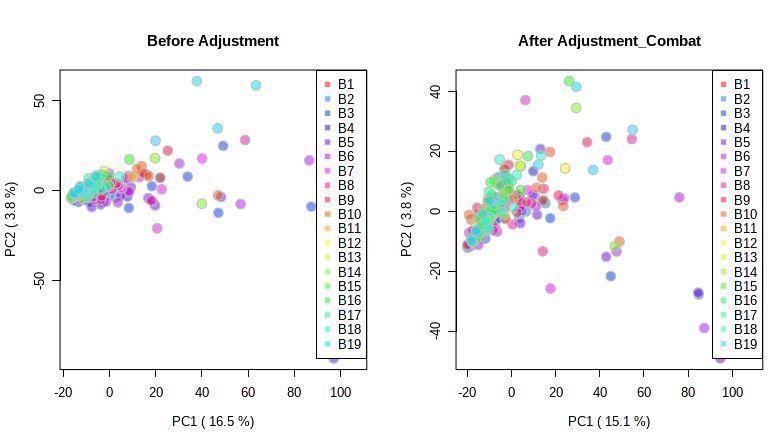


*Figure S1: Principal component analysis (PCA) of all patients’ samples, grouped according to the batches they were analysed in, before and after batch correction using the COMBAT function. This output indicates no prominent batch effect* (cumulative variation *explained by the first two principal components (PCs) was 20.3 % before and 18.9% thereafter). B = batch*.

*
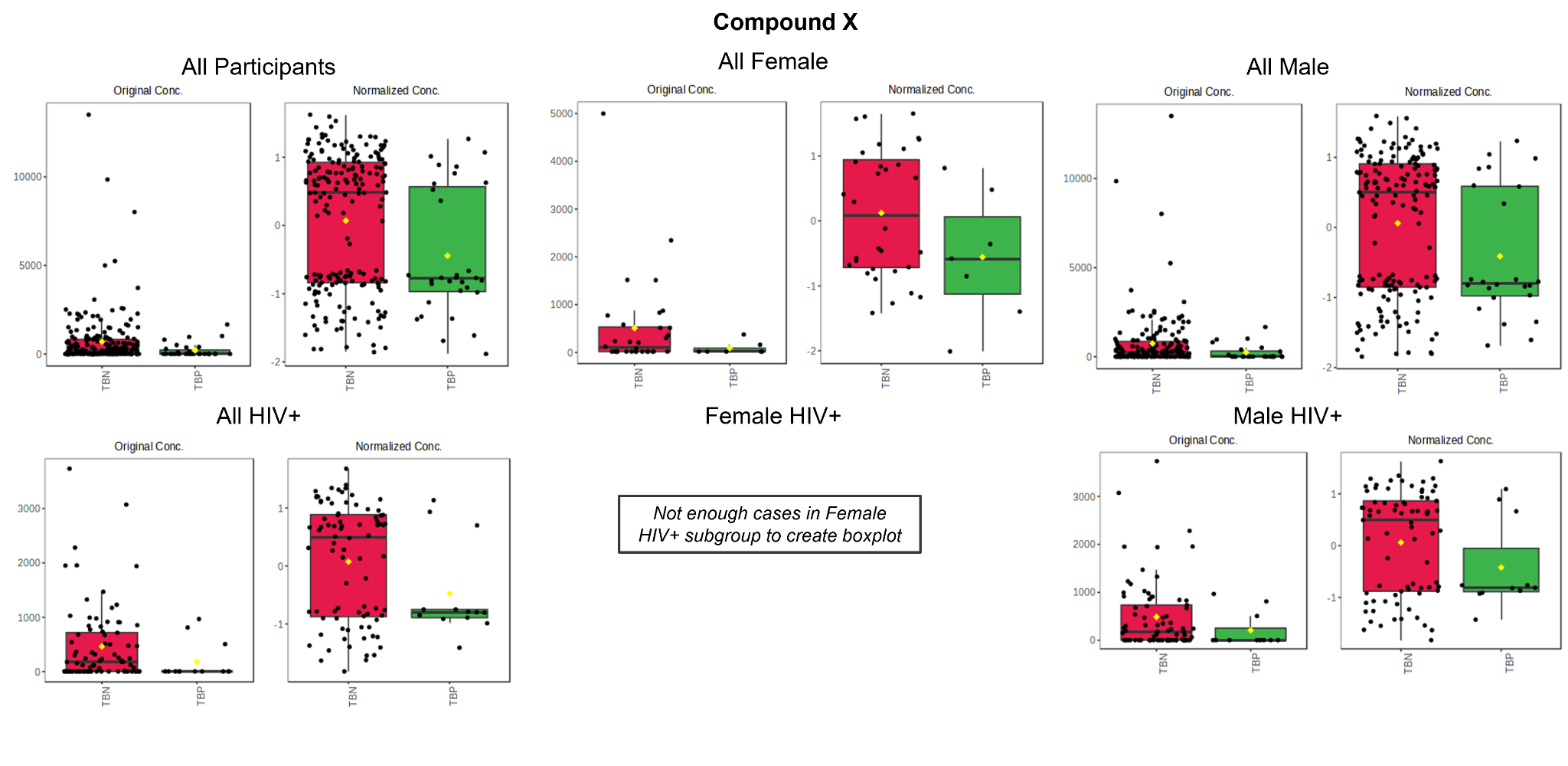
*

*Figure S2:* Boxplots of compound X in all the subgroup comparisons, compiled using the original concentrations and normalised concentrations. Due to the small sample size of the female HIV+ group, boxplots could not be created.


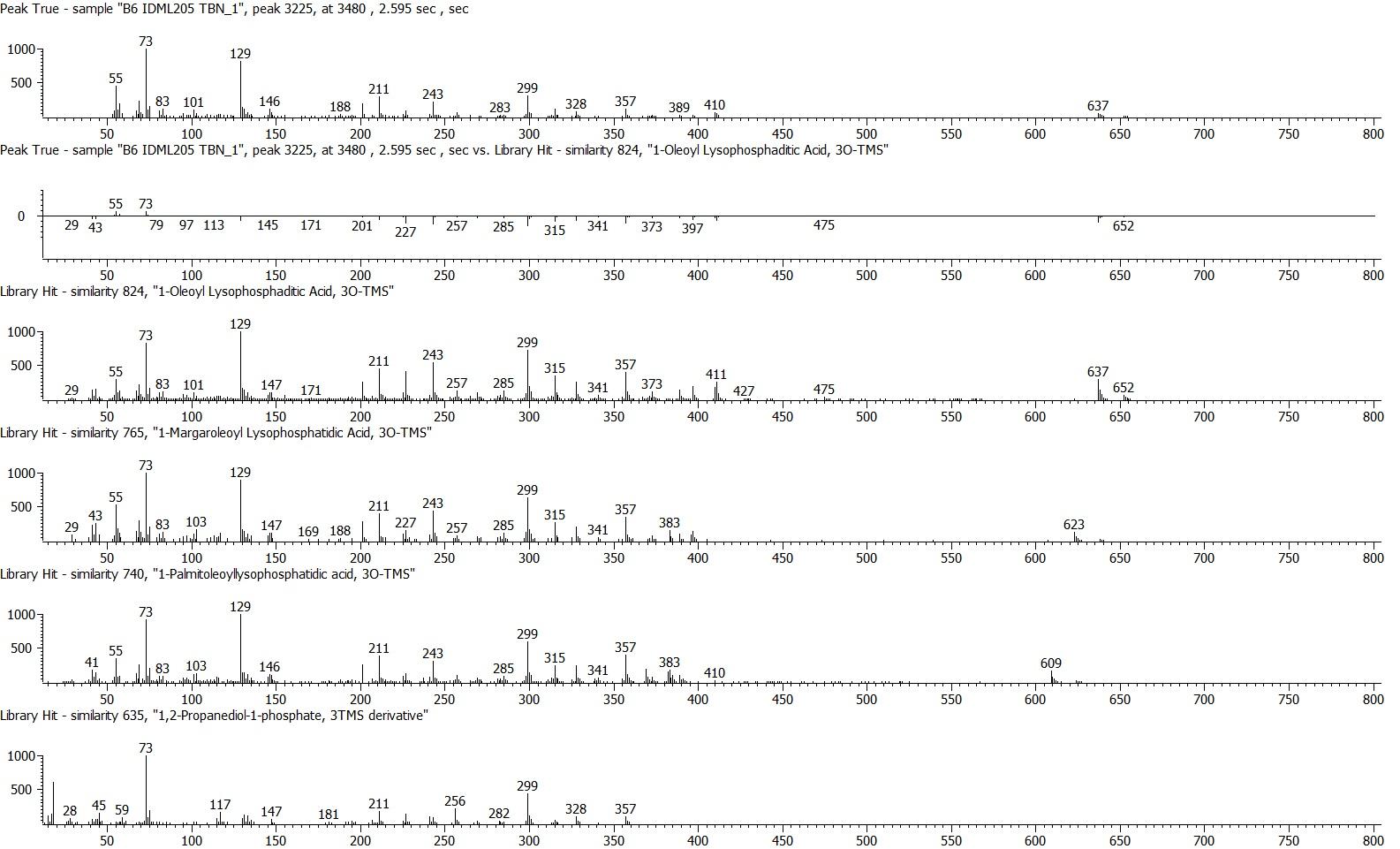


*Figure S3: Mass spectrum of the compound detected as statistically significant in all subgroup comparisons (top), compared to the mass spectrum in the NIST library (bottom), leading to the annotation of the compound as 1-oleoyl lysophosphaditic acid (LPA) based on a 82.4% similarity. The differences between the two mass spectra are given as the middle figure.*


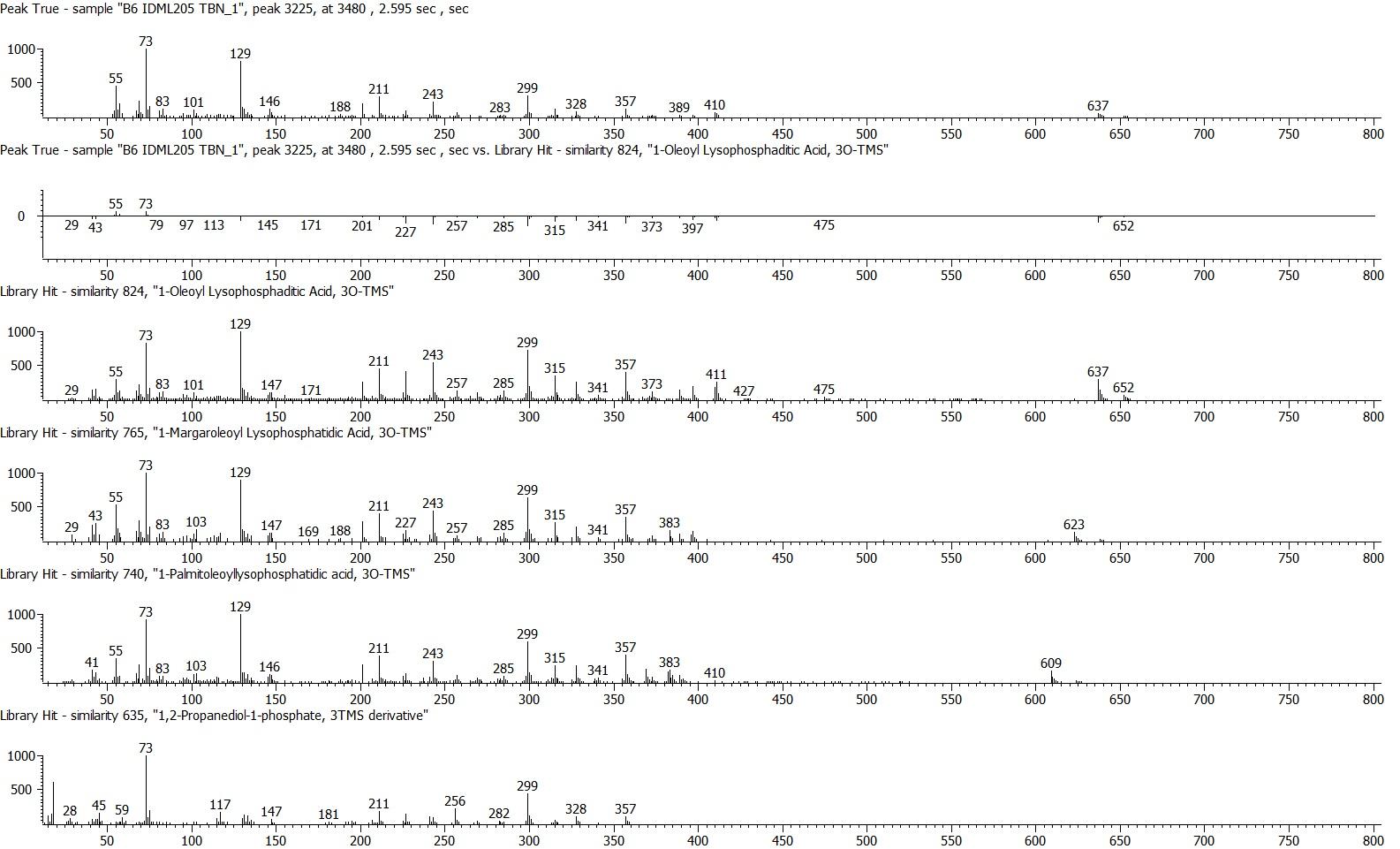


*Figure S4: NIST library mass spectra of 1-margaroleoyl LPA and 1-palmitoleoyl and LPA*


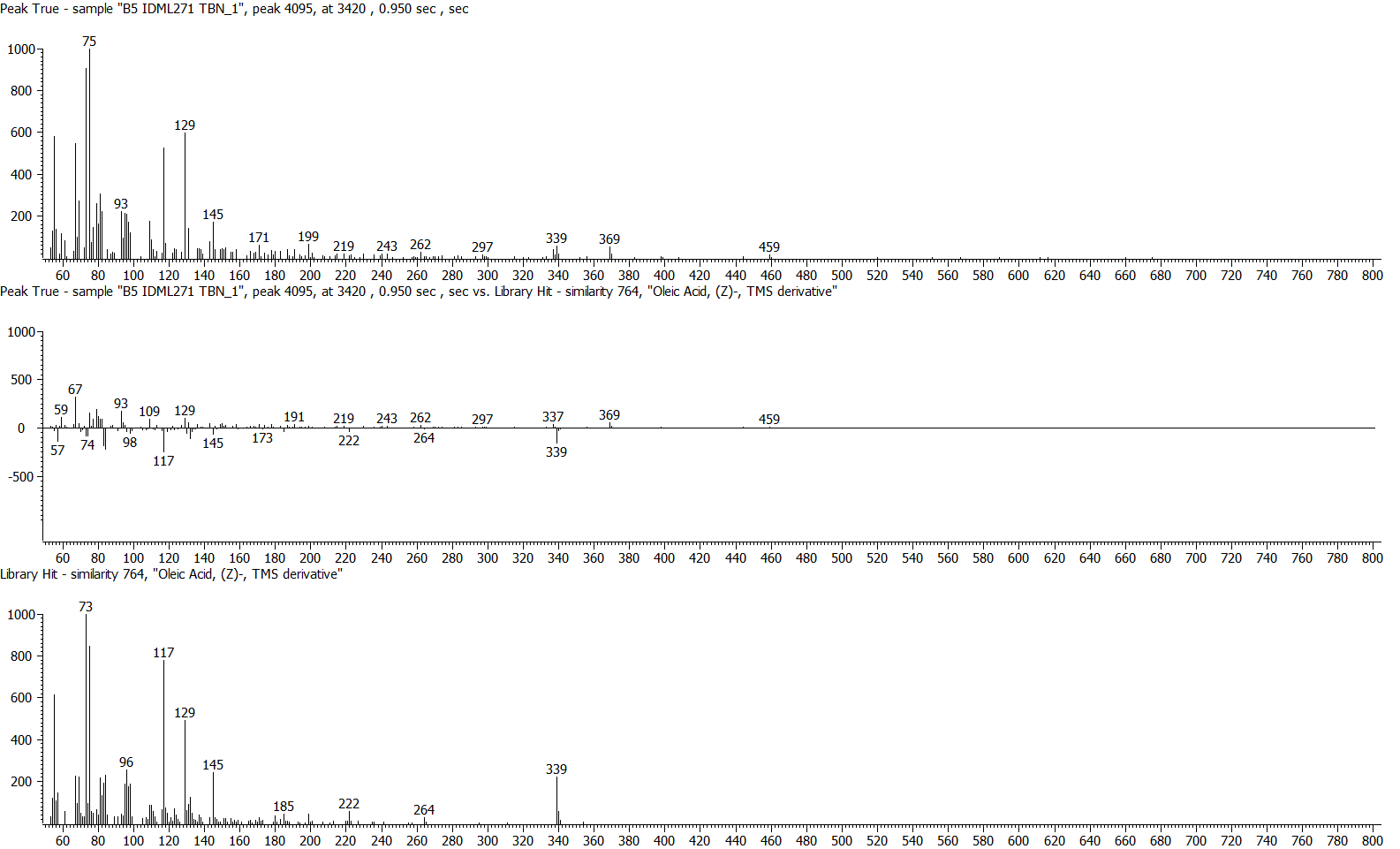


*Figure S5: Mass spectrum of the unannotated compound detected as statistically significant in all subgroup comparisons (top), compared to the best matched (76,4% similarity) mass spectrum in the NIST library (bottom), leading to the annotation of the compound as oleic acid. The differences between the two mass spectra are given as the middle figure.*
